# β-Elemene promotes ferroptosis and reverses radioresistance in gastric cancer by inhibiting the OTUB1-GPX4 interaction

**DOI:** 10.3389/fphar.2024.1469180

**Published:** 2024-10-17

**Authors:** Jiancheng He, Ming Li, Jiapeng Bao, Yifeng Peng, Wanjiang Xue, Junjie Chen, Jun Zhao

**Affiliations:** ^1^ Department of Gastrointestinal Surgery, Affiliated Hospital and Medical School of Nantong University, Nantong, China; ^2^ Research Center of Clinical Medicine, Affiliated Hospital and Medical School of Nantong University, Nantong, China; ^3^ Nantong Key Laboratory of Gastrointestinal Oncology, Nantong, China; ^4^ Department of Pediatric Surgery,Affiliated Hospital and Medical School of Nantong University, Nantong, China

**Keywords:** gastric cancer, β-elemene, ferroptosis, ubiquitination, GPx4, OUTB1

## Abstract

**Introduction:**

β-Elemene, derived from *Curcuma zedoaria* (Wenyujin), is clinically recognized for inducing apoptosis, inhibiting cell cycle progression, and reversing chemotherapy resistance in various cancers. However, its effects on radioresistant gastric cancer (GC) remain unclear.

**Methods:**

In this study, radioresistant GC cell lines (MKN45/IR and AGS/IR) were established via multiple low-dose radiations. The impact of β-elemene on radiosensitivity was assessed using CCK-8 and clonogenic assays, with ferroptosis markers such as ROS, MDA, and Fe^2+^ levels measured. Additionally, the influence of β-elemene on GPX4 and its interaction with OTUB1 was examined through qRT-PCR, Western blot, immunofluorescence, co-immunoprecipitation, and in vivo studies.

**Results:**

Our findings indicate that β-elemene reverses radioresistance in GC cells and significantly inhibits cell growth when combined with radiotherapy. β-Elemene treatment elevated ROS, MDA, and Fe^2+^ levels, enhancing ferroptosis, which was confirmed by Ferrostatin-1 and Deferoxamine inhibition studies. Mechanistic analysis revealed that β-elemene disrupts the OTUB1-GPX4 interaction, leading to increased GPX4 ubiquitination and degradation, thus promoting ferroptosis. *In vivo* studies further demonstrated that β-elemene combined with radiotherapy significantly suppressed tumor growth compared to radiotherapy alone.

**Discussion:**

These results suggest that β-elemene effectively modulates radioresistance in GC by targeting the GPX4 pathway and inducing ferroptosis. This highlights its potential as a therapeutic adjunct in radiotherapy for resistant GC cases.

## 1 Introduction

Gastric cancer (GC) is among the most aggressive malignancies, ranking fifth in global incidence and third in cancer-related mortality worldwide (Ding et al., 2023; Qu et al., 2023). Despite advances in diagnosis and treatment, the prognosis for patients with advanced GC remains poor (Smyth et al., 2020). Radiotherapy, a cornerstone of GC treatment, induces various forms of DNA damage; however, its effectiveness is often hampered by the development of radioresistance (Meng and Lu, 2024; Varghese et al., 2024). As a result, overcoming this resistance to enhance radiotherapy efficacy has become a central focus of research.

Emerging evidence indicates that ferroptosis, an iron-dependent form of cell death characterized by lipid peroxidation, can inhibit the growth of malignant tumors resistant to conventional therapies (Wang et al., 2023; He et al., 2023; Luo et al., 2023). For example, photodynamic therapy (PDT) significantly reduces oral squamous cell carcinoma growth by increasing ROS levels and inducing ferroptosis (Zhu et al., 2019). Similarly, radiotherapy has been shown to trigger substantial ferroptosis, contributing to its anticancer effects. In KEAP1-deficient lung cancer cells, ferroptosis and radioresistance are mediated by the CoQ-FSP1 axis, with sensitization achieved through inhibiting this pathway (Koppula et al., 2022). Unlike apoptosis, necrosis, and autophagy, ferroptosis has unique genetic, biochemical, and morphological characteristics (Zhang et al., 2019). Clinically, combining radiotherapy with chemotherapy, targeted therapy, or immunotherapy is often necessary to effectively target cancer cells (Wang et al., 2018). Notably, the success of these treatments may also relate to the induction of ferroptosis, positioning it as a vital strategy in overcoming drug resistance.

Natural products have long been recognized as promising therapeutic agents, with numerous studies highlighting their potential to sensitize tumor cells to radiation via key molecular mechanisms (Komorowska et al., 2022). Among these, β-elemene, an anti-cancer compound extracted from the Zingiberaceae plant Curcuma zedoaria, stands out. Known chemically as 1-methyl-1-vinyl-2,4-diisopropylcyclohexane, β-elemene has been extensively used in treating various malignancies, including lung, bladder, leukemia, ovarian, and glioblastoma cancers (Wang et al., 2005; Li et al., 2013a; Yu et al., 2011; Li et al., 2013b; Yao et al., 2008). Research has demonstrated β-elemene’s capability to reverse drug resistance and enhance the effects of chemotherapy (Liu et al., 2015). Recent findings also suggest its role as a radiosensitizer, promoting DNA damage and inhibiting repair, and increasing apoptosis in treated lung cancer cells (Zou et al., 2020). Yet, its impact on ferroptosis and the radiosensitivity of GC cells resistant to radiation remains unexplored.

In this study, we aim to investigate the effects of β-elemene on radioresistant GC cells, focusing on its potential to induce ferroptosis and enhance radiosensitivity. By elucidating the underlying mechanisms, we hope to establish β-elemene as a potent therapeutic adjunct in the treatment of radioresistant GC, ultimately improving patient outcomes.

## 2 Materials and methods

### 2.1 Cell culture and radiation treatment

Human GC cell lines MKN-45 were obtained from BeNa Culture Collection (Shanghai, China), AGS from the Cell Bank of the Chinese Academy of Sciences (Shanghai, China). All cell lines were cultured in 1,640 medium (Gibco, United States) supplemented with 10% fetal bovine serum (Clark, Shanghai, China) and penicillin-streptomycin (Gibco, United States), in a humidified environment of 37°C with 5% CO2. To establish radioresistant cell lines, AGS and MKN-45 cells were irradiated at a cell density of 60% with a dose of 4 Gy. Immediately following irradiation, the culture medium was replaced with fresh medium, which was then changed every 2 days. Cells were continuously cultured until they reached 90% confluence, at which point they were enzymatically dissociated using trypsin and transferred to new T25 culture flasks. This process of culturing and irradiation was repeated until a cumulative dose of 60 Gy was achieved (over 15 sessions), resulting in the establishment of the radioresistant cell lines MKN-45/IR and AGS/IR (Li et al., 2014; Petragnano et al., 2020).

### 2.2 Cell counting Kit-8 (CCK-8), clone formation assay and EDU assay

Cell viability was assessed using the CCK-8 according to the manufacturer’s instructions. Cells were seeded in 96-well plates at a density of 1,000 cells per well. To prevent edge effects, equal volumes of PBS were added to the peripheral wells. Cells were then subjected to irradiation at doses of 0, 2, 4, 6, and 8 Gy or treated with 100 mg/L β-elemene, or a combination of both. After 48 h, 100 μL of diluted CCK-8 solution was added to each well and incubated for 1–2 h. Absorbance was measured at 450 nm using a microplate reader. For the clonogenic assay, logarithmically growing cells were used. After treatment with trypsin to create a single-cell suspension, cells were seeded in 6-well plates at a density of 1,000 cells per well. Twenty-4 hours later, cells were either irradiated at varying doses of 0, 2, 4, 6, and 8 Gy, treated with 100 mg/L β-elemene, or received both treatments. After 14 days of incubation, colonies were fixed with polyethylene glycol for 20 min and stained with crystal violet. Colonies containing at least 50 cells were counted. Colony survival rates for each treatment were calculated. The EDU assay was performed using the BeyoClick™ EdU-555 Cell Proliferation Assay Kit (Beyotime, Shanghai, China) according to the manufacturer’s instructions. Images were captured using a Leica Thunder fully automated inverted microscope. The experiment was conducted in triplicate.

### 2.3 Assessing β-elemene mechanisms with inhibitors

To explore how β-elemene inhibits the proliferation of radioresistant GC, several specific inhibitors were utilized. These included the ferroptosis inhibitor Ferroptosis Inhibitor-1 (Fer-1), the iron chelator Desferrioxamine (DFO), the autophagy inhibitor 3-Methyladenine (3-MA), the apoptosis inhibitor Z-VAD-FMK (Z-Val-Ala-Asp(OMe)-fluoromethylketone), and the necrosis inhibitor Necrostatin-1 (Nec-1). All reagents were sourced from MedChemExpress (the United States) and stored at −20°C.

### 2.4 Quantitative real-time PCR (qRT-PCR) assay

RNA was isolated utilizing TRIzol reagent (Invitrogen, United States). The qRT-PCR process was carried out in accordance with methods outlined earlier (Sindhuja et al., 2022). Primer sequences are provided: GPX4:F:5′-GAGGCAAGACCGAAGTAAACTAC-3′,R:5′-CCGAACTGGTTACACGGGAA-3’.

### 2.5 Malondialdehyde (MDA) assay

Operation was performed according to the instructions of the MDA assay kit (Beyotime, China). Prepare the working solution by washing the cells to be tested three times with PBS buffer solution. Then, lyse the cells with RIPA lysis buffer containing 1% PMSF for 30 min. Transfer 0.15 mL of the lysate to the sample tube. Add 0.15 mL of the standard solution to the standard tube and 0.15 mL of distilled water to the blank tube. Add 2.5 mL of TBA to each tube, incubate at 100°C for 40 min, and cool with tap water. Centrifuge at 4,000 rpm for 10 min. Transfer the supernatant to a 96-well plate, set the wavelength to 532 nm, and measure the absorbance using a multifunctional microplate reader.

### 2.6 Reactive oxygen species (ROS) measurement

Intracellular ROS levels were measured using 2,7-dichlorofluorescein diacetate (DCFH-DA) (Yeasen, Shanghai, China). Cells were incubated with 10 μM DCFH-DA diluted in RPMI-1640 medium at 37°C in the dark for 30 min. Fluorescence intensity was quantified using ImageJ software. Cells were analyzed using flow cytometry.

### 2.7 Iron ion detection

Operate according to the instructions of the iron ion colorimetric test kit manual (Beyotime, China).

### 2.8 Western blot assay

Total protein separation and Western blot were conducted following the aforementioned methods (Liu et al., 2020; He et al., 2024). The following antibodies were utilized: anti-GPX4, anti-GAPDH, anti-HA and anti-Flag (Proteintech, China), anti-OTUB1(Affinity, China).

### 2.9 Co-immunoprecipitation assay (Co-IP)

Total cell lysates were incubated with 1 μg of primary antibody or negative control rabbit IgG overnight at 4°C. Subsequently, 20 μL of Protein A+ G Agarose (Bioworld Technology, St. Louis Park, MN, United States) was added, and the mixture was further incubated for 2 h at 4°C. The protein-antibody mixtures were washed four times with PBS and collected by agarose bead spinning. Following this, proteins were identified through SDS-polyacrylamide gel electrophoresis and subsequent Western blot analysis.

### 2.10 Immunofluorescence assay (IF)

First, GC cells were washed three times with cold phosphate-buffered saline (PBS) (Gibco, United States). They were then fixed with 4% paraformaldehyde (Beyotime, China) for 25 min, followed by permeabilization with 0.1% Triton X-100 (Beyotime, China) for 10 min. This was followed by a blocking step in 5% bovine serum albumin (Solarbio, China). Subsequently, the cells were incubated with anti-GPX4 antibody overnight at 4°C, followed by three washes with PBS. Next, the cells were incubated with Alexa Fluor 488 conjugated goat anti-Mouse IgG (ABclonal, China) for 1 h, followed by three washes with PBS. The cells were then incubated with anti-OTUB1 antibody overnight at 4°C, followed by PBS washes. Subsequently, the cells were incubated with Alexa Fluor 647 conjugated goat anti-Rabbit IgG (ABclonal, China) for 1 h and stained with DAPI (Cell Signaling Technology, United States) for 15 min. Imaging of the stained cells was performed using a Zeiss LSM 900 confocal microscope.

### 2.11 Immunohistochemistry (IHC)

IHC were performed according to methods previously described (Zhu et al., 2021; Hu et al., 2020; Yilin Hu et al., 2024). Anti-GPX4, anti-Ki-67, anti-4-HNE antibodies were used. Two experienced pathologists manually scored the staining intensity according to the following criteria: 0 = no staining, 1 = weak staining, 2 = moderate staining, 3 = strong staining. The proportion of positive cells was scored as 1 (0%–10%), 2 (11%–50%), 3 (51%–80%), 4 (81%–100%) (Lu et al., 2020; Zhu et al., 2019; Xian et al., 2014; Jing-Lin Wang et al., 2023). The final IHC score was calculated by multiplying the intensity score by the percentage of positive cells.

### 2.12 Animal experiment

Thirty-two 4-week-old male nude mice (purchased from the Model Animal Research Center of Nantong University) were randomly divided into eight groups. Four groups were injected with MKN-45/IR cells (control group, β-elemene group, radiation therapy group, and combination therapy group), and four groups were injected with AGS/IR cells (control group, β-elemene group, radiation therapy group, and combination therapy group), with four mice per group. Each mouse was subcutaneously injected in the right axillary area with 200 μL of MKN-45/IR or AGS/IR cells (2 × 10^6 cells). The growth of the mice was monitored regularly, and once the subcutaneous tumor size reached approximately 150 mm³ (around 15 days), different treatments were administered to the groups. Tumor volume and weight were measured every 3 days. After 36 days, the mice were euthanized, and tumor tissues were harvested, weighed, photographed, recorded, and tumor growth curves were plotted. All animal experiments were approved by the Animal Ethics Committee of Nantong University.

### 2.13 Statistical analysis

Data were analyzed using GraphPad Prism 8.0 and the R programming language (version 4.2.3). Measurement data with normal distribution were expressed as mean ± SD and statistically analyzed with two-sample *t*-test. Non-normal distribution measurement data were expressed as median (range) and analyzed by Mann-Whitney U test. For groups of three or more that conform to a normal distribution, one-way ANOVA is utilized, while for those that do not follow a normal distribution, the Kruskal–Wallis test is employed. All statistical analyses were two-sided, and *p* < 0.05 was used to define statistical significance. All experiments were conducted more than three times.

## 3 Results

### 3.1 β-Elemene treatment reverses the radioresistance of GC cells

**FIGURE 1:**
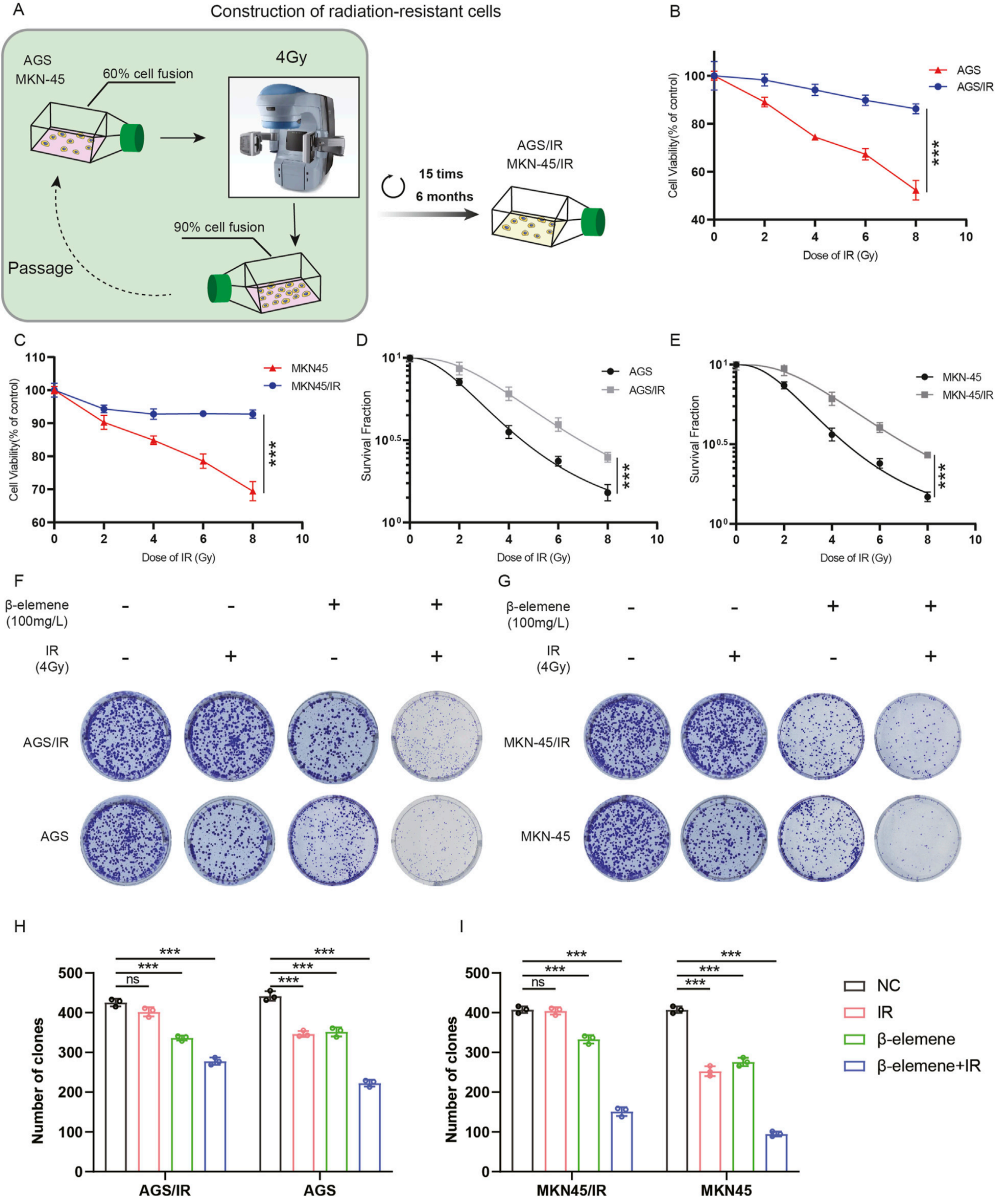
β-Elemene treatment reverses the radioresistance of GC cells. **(A)** Construction of radioresistant GC cell line models. **(B–C)** Evaluation of radiosensitivity in radioresistant GC cell lines MKN-45/IR and AGS/IR using the CCK-8 assay. **(D–E)** Assessment of colony formation following treatment with various radiation doses; colonies counted 2 weeks post-treatment. **(F–I)** Investigation of the impact of β-elemene and/or 4 Gy radiation exposure on the proliferative capacity of GC cells via colony formation.***p < 0.001.

To investigate whether β-elemene treatment can reverse the radioresistance of GC cells, we first established radioresistant GC cell lines (MKN-45/IR and AGS/IR) (Figure 1A). The radioresistance of MKN-45/IR and AGS/IR cells was assessed using the Cell Counting Kit-8 (CCK-8) assay and clonogenic survival assay. Results showed that MKN-45/IR and AGS/IR cells exhibited higher growth capabilities and survival rates compared to their parental cells (Figures 1B–E). We further determined the mean lethal dose (D0), quasi-threshold dose (Dq), and the sensitization enhancement ratio (SER) of cell survival before and after radiotherapy. The results indicated that the D0 for MKN-45 was 3.130 and the Dq was 2.665, while for MKN-45/IR cells, the D0 was 4.476 and the Dq was 3.052, with an SER of 0.75. For AGS, the D0 was 3.302 and the Dq was 2.376, while for AGS/IR cells, the D0 was 4.441 and the Dq was 2.904, with an SER of 0.74. These findings suggest that the resistant cell lines exhibit stronger radioresistance than their parental cells. To further explore the impact of β-elemene treatment on radioresistance, we validated this effect using a clonogenic formation assay. The results demonstrated that the combination of β-elemene and radiotherapy significantly reduced the colony-forming ability of MKN-45/IR and AGS/IR cells (Figures 1F–I).

### 3.2 β-Elemene treatment can induce ferroptosis in radioresistant GC cells

**FIGURE 2:**
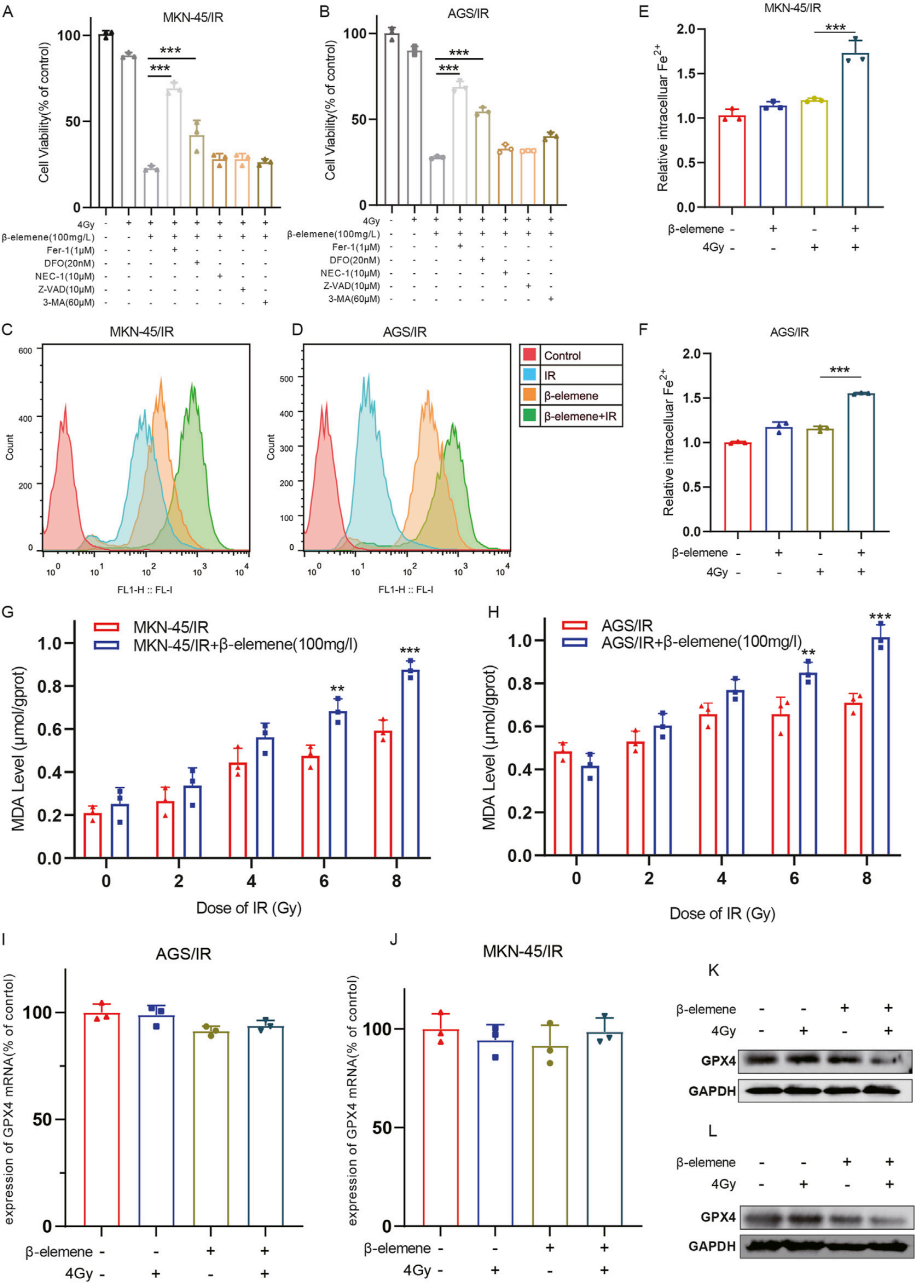
β-Elemene treatment can induce ferroptosis in Radioresistant GC cells. **(A–B)** MKN-45/IR and AGS/IR cells were treated for 48 h with 4 Gy irradiation and/or β-elemene (100 mg/L) combined with various cell death inhibitors, followed by cell viability assessment using the CCK-8 assay. **(C–D)** The levels of reactive oxygen species (ROS) in MKN-45/IR and AGS/IR cells were measured 48 h after treatment with β-elemene (100 mg/L) and/or irradiation (4 Gy) using flow cytometry. **(E–F)** The intracellular levels of Fe2+ in MKN-45/IR and AGS/IR cells were determined 48 h post-treatment with β-elemene (100 mg/L) and/or radiation (4 Gy) using an Fe2+ detection assay kit. **(G–H)** The levels of malondialdehyde (MDA) in MKN-45/IR and AGS/IR cells were analyzed 48 h after treatment with β-elemene (100 mg/L) and/or various doses of radiation. **(I–L)** The expression of GPX4 in AGS/IR and MKN-45/IR cells was evaluated 48 h post-treatment with β-elemene (100 mg/L) and/or radiation (4 Gy) using qRT-PCR and Western blot. **p < 0.01, ***p < 0.001.

To determine the primary molecular mechanisms by which β-elemene combined with radiotherapy inhibits the growth of radioresistant GC cells, cells were first pre-treated with various cell death inhibitors. Cells were treated with the ferroptosis inhibitor Fer-1, the iron chelator DFO, the autophagy inhibitor 3-methyladenine (3-MA), the apoptosis inhibitor Z-VAD, and the necroptosis inhibitor Necrostatin-1. Cell survival levels were assessed 48 h later. The results indicated that both Fer-1 and DFO significantly reduced the cell death induced by the combination of radiotherapy and β-elemene (Figures 2A,B). This suggests that ferroptosis may play a key role in β-elemene’s ability to reverse radiotherapy resistance. Subsequently, to further validate this hypothesis, we measured levels of reactive oxygen species (ROS), iron ions, and lipid peroxidation in MKN-45/IR and AGS/IR cells. Consistent with previous findings, these indicators were significantly elevated after the combined treatment of β-elemene and radiotherapy (Figures 2C–H). To explore the role of ferroptosis in β-elemene’s reversal of radiotherapy resistance in GC cells, we focused on a key ferroptosis regulatory protein, Glutathione Peroxidase 4 (GPX4). GPX4 is the principal enzyme that prevents the accumulation of peroxidized lipids in cells, and its activity is closely linked to ferroptosis (Liu et al., 2024). Therefore, we assessed the expression of GPX4 in radioresistant GC cells treated with β-elemene via qPCR and Western blot. Although there was no significant change in GPX4 mRNA expression levels, the protein levels decreased significantly, suggesting that β-elemene may regulate the expression of GPX4 by affecting protein stability or degradation pathways, thereby further promoting ferroptosis (Figures 2I–L).

### 3.3 β-Elemene promotes the ubiquitination and degradation of GPX4 in radioresistant GC cells

**FIGURE 3:**
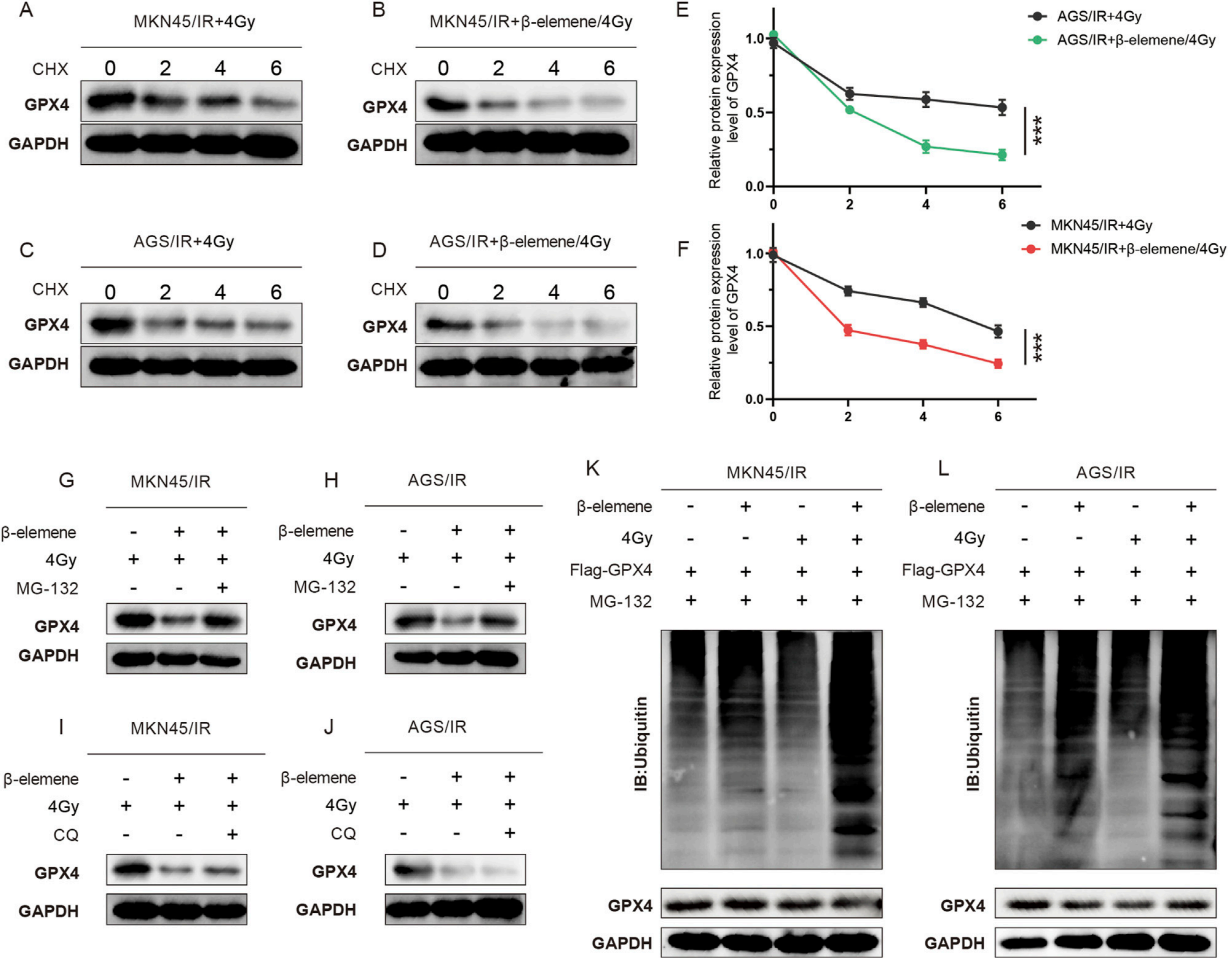
β-Elemene promotes the ubiquitination and degradation of GPX4 in Radioresistant GC cells. **(A–F)** Western blot analysis of GPX4 protein levels in MKN-45/IR and AGS/IR cells treated with radiation (4 Gy) with or without β-elemene (100 mg/L). Cells were harvested at 0, 2, 4, and 6 h after treatment with CHX. **(G–J)** Western blot analysis of GPX4 protein levels in MKN-45/IR and AGS/IR cells treated with radiation (4 Gy) with or without β-elemene (100 mg/L). Cells were collected at 0, 2, 4, and 6 h after treatment with either MG-132 or CQ. **(K–L)** Investigation of ubiquitination levels of GPX4 in cells co-transfected with Ub plasmid, Flag-GPX4, and MG132, treated with radiation (4 Gy) with or without β-elemene (100 mg/L).

To further clarify how β-elemene affects GPX4 protein levels, we initially treated cells with cycloheximide (CHX) to block new protein synthesis, allowing us to observe the dynamics of protein degradation. Experimental results indicated that in the combination of β-elemene and radiotherapy, GPX4 degraded faster than in the radiotherapy-alone group (Figures 3A–F). This suggests that β-elemene may enhance the degradation of GPX4. Considering that the ubiquitin-proteasome pathway and the autophagy-lysosome pathway are two major routes regulating protein degradation, we used the proteasome inhibitor MG132 and the autophagy inhibitor chloroquine (CQ) to further explore the role of these pathways. Treatment with MG132 significantly inhibited the degradation of GPX4, while treatment with CQ had little effect on the degradation of GPX4 (Figures 3G–J). This result indicates that under the conditions of combined β-elemene and radiotherapy, the degradation of GPX4 primarily occurs through the ubiquitin-proteasome pathway. To verify this, we further examined the ubiquitination levels of GPX4. Compared to the radiotherapy-alone group, the ubiquitination levels of GPX4 were significantly increased in the β-elemene and radiotherapy combination group, supporting our hypothesis that β-elemene may act by promoting the ubiquitination and subsequent proteasome-mediated degradation of GPX4 (Figures 3K, L).

### 3.4 β-Elemene inhibits the interaction between OTUB1 and GPX4 in radioresistant GC cells

**FIGURE 4:**
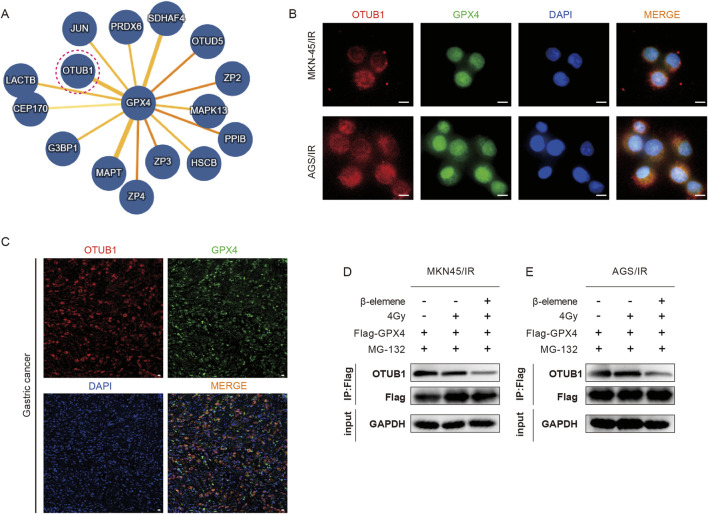
β-Elemene inhibits the interaction between OTUB1 and GPX4 in Radioresistant GC cells. **(A)** Potential interacting proteins of GPX4 predicted using the IntAct online database. **(B–C)** IF experiments and mIHC validate the colocalization of GPX4 and OTUB1 in MKN-45/IR and AGS/IR cells. Scale bar, 10 μm. **(D–E)** Co-immunoprecipitation (Co-IP) assays to detect the interaction between GPX4 and OTUB1 in MKN-45/IR and AGS/IR cells treated with radiation (4 Gy) with or without β-elemene (100 mg/L).

In order to delve deeper into the mechanism of GPX4 ubiquitination and degradation, we utilized the IntAct online database to predict potential interacting proteins of GPX4 (Figure 4A). Among these proteins, OTUB1 has been identified as a deubiquitinase that is intricately involved in the ubiquitin-dependent protein degradation pathway. Previous studies have demonstrated that in GC cell lines, OTUB1 can form a complex with GPX4, thereby reducing the ubiquitination levels of GPX4 and inhibiting its proteasomal degradation (Li et al., 2023). We also found this phenomenon in radioresistant GC cell lines. IF assays confirmed the co-localization of OTUB1 with GPX4 (Figures 4B,C), and COIP assays showed that the interaction between GPX4 and OTUB1 was robust in both the control and the radiotherapy-alone groups. However, this interaction was significantly diminished in the group treated with a combination of β-elemene and radiotherapy (Figures 4D, E).

### 3.5 β-elemene can reverse GC cell radioresistance through ferroptosis *in vivo*

**FIGURE 5:**
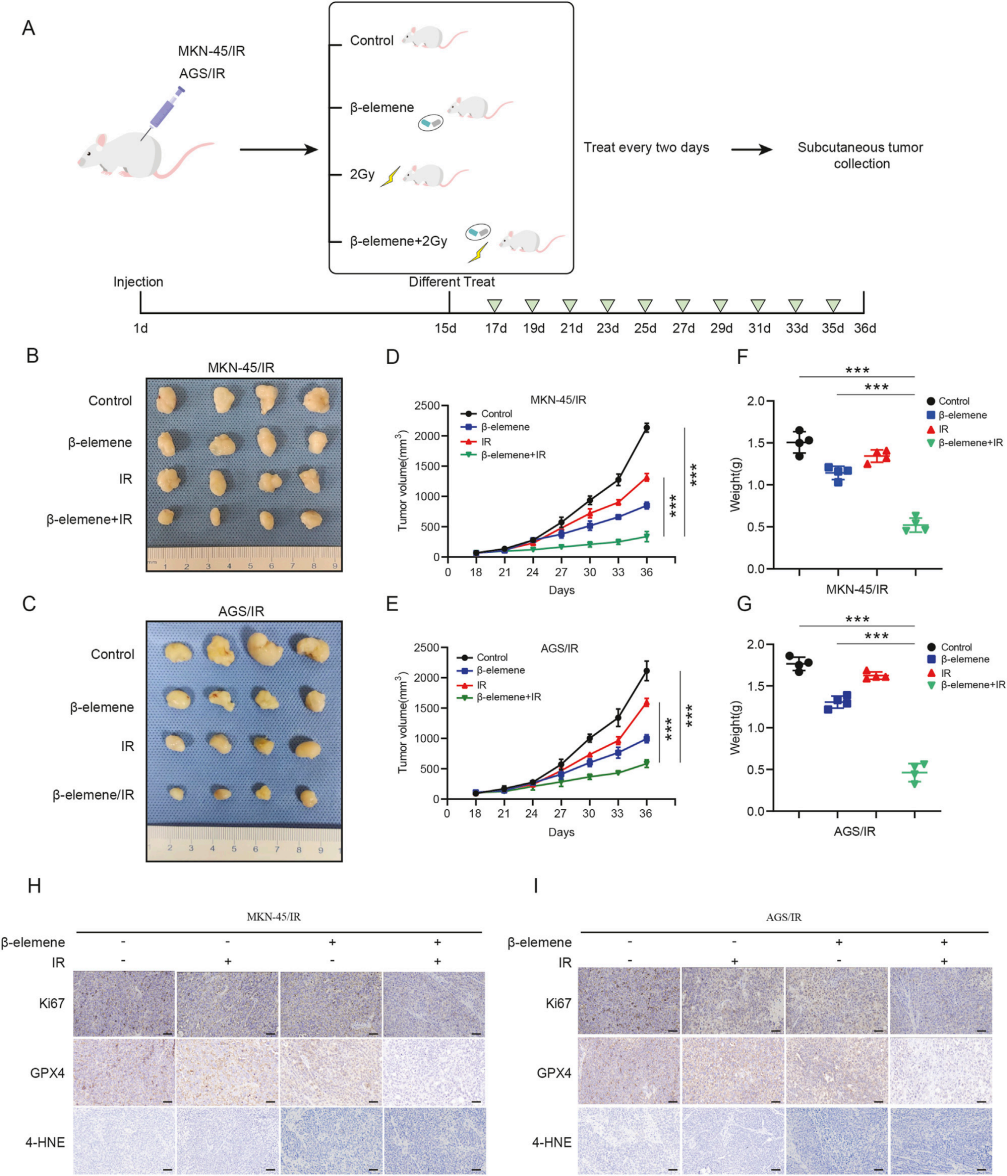
β-elemene can reverse GC cell radioresistance through ferroptosis *in vivo*. **(A)** Flowchart for constructing a nude mouse model of subcutaneous tumors treated with β-elemene combined with radiotherapy. **(B–C)** Typical images of subcutaneous xenograft tumors. **(D–G)** Volume and weight of subcutaneous xenograft tumors (n = 4). **(H–I)** IHC staining of subcutaneous tumors from different groups. Scale bar, 50 μm ***p < 0.001.

To explore the therapeutic potential of β-elemene combined with radiotherapy *in vivo*, we established a subcutaneous tumor model in nude mice by injecting MKN-45/IR or AGS/IR cells subcutaneously (Figure 5A). Once tumors developed, treatments were administered either with β-elemene via gavage (0.2 mL per administration), radiotherapy alone (2 Gy per administration), or a combination of both. Results indicated that tumor growth in the nude mice treated with the combination of β-elemene and radiotherapy was significantly inhibited (Figures 5B–G). Immunohistochemical staining of the tumor tissues showed elevated levels of 4-HNE in the combination therapy group, whereas expressions of Ki-67 and GPX4 were lower compared to the control group (Figures 5H, I).

## 4 Discussion

**FIGURE 6:**
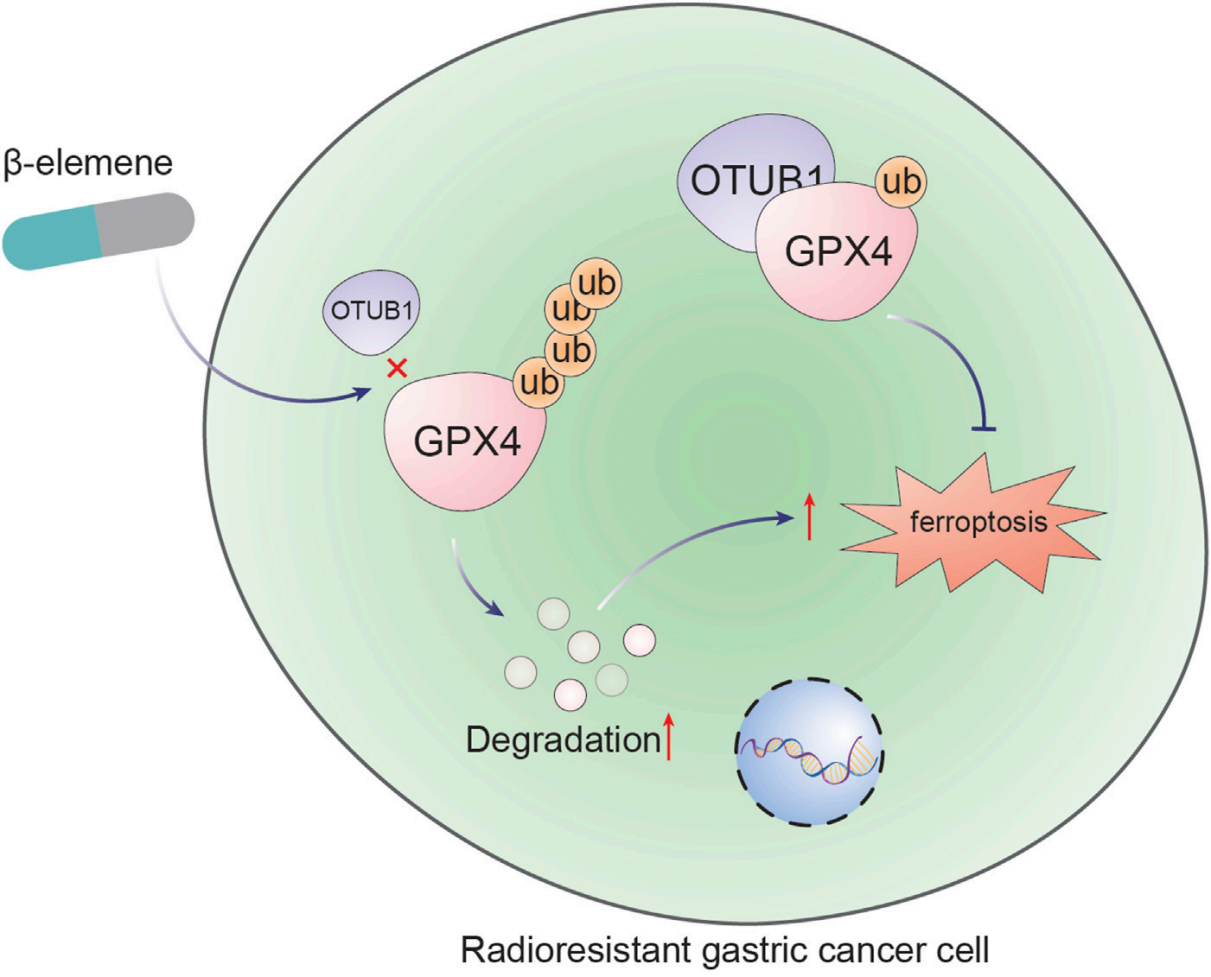
β-Elemene promotes ferroptosis and reverses radioresistance in gastric cancer by inhibiting the OTUB1-GPX4 interaction.

This study elucidates the capacity of β-elemene to reverse radioresistance in GC through the modulation of ferroptosis. Specifically, it has been demonstrated that β-elemene inhibits the interaction between OTUB1 and GPX4, facilitating the ubiquitin-mediated degradation of GPX4, enhancing ferroptosis, and consequently augmenting the radiosensitivity of GC cells (Figure 6). These findings provide a novel perspective on the therapeutic use of β-elemene to overcome radioresistance in GC treatment.

Recent studies have demonstrated the potential of β-elemene as a radiosensitizer across various cancer types. For instance, research in non-small-cell lung cancer showed that β-elemene enhances radiosensitivity by inhibiting EMT and reducing cancer stem cell traits through the Prx-1/NF-κB/iNOS signaling pathway (Zou et al., 2020). Similarly, β-elemene has been reported to promote DNA damage and inhibit repair mechanisms, resulting in increased apoptosis in glioblastoma and bladder cancer cells (Li et al., 2011). In glioblastoma, β-elemene’s ability to enhance both radiosensitivity and chemosensitivity was linked to its inhibition of the ATM signaling pathway (Liu et al., 2015). Our study revealing that β-elemene reverses radioresistance in GC cells primarily by promoting ferroptosis through the inhibition of the OTUB1-GPX4 interaction. This mechanism underscores the unique radiosensitizing capabilities of β-elemene and its potential to improve radiotherapy outcomes in GC.

GPX4 is an important negative regulator of ferroptosis (Chen et al., 2023). The primary intracellular function of GPX4 is to scavenge peroxidized lipids from cell membranes, preventing peroxidative damage (Forcina and Dixon, 2019). It converts reduced glutathione (GSH) to oxidized glutathione (GSSG) while reducing peroxidized lipids to non-toxic lipids. Thus, it plays a crucial role in inhibiting ferroptosis (Xue et al., 2023). In oncology, GPX4 is recognized for its critical role in mitigating lipid peroxidation and averting ferroptosis, thereby supporting cancer cell survival under therapeutic stress.

Recent studies have found that deubiquitinases selectively regulate tumor-associated proteins closely related to tumorigenesis and development (Dewson et al., 2023). OTU domain-containing ubiquitin aldehyde-binding protein B1 (OTUB1) is a member of the DUB family, widely expressed in the kidney, intestine, brain, liver, and lung (Nijman et al., 2005). OTUB1 is a deubiquitinating enzyme involved in the malignant progression of tumors (Liao et al., 2022). For example, OTUB1 promotes colorectal cancer metastasis by promoting EMT and serves as a potential distant metastasis marker in colorectal cancer (Saldana et al., 2019). OTUB1 also promotes esophageal squamous cell carcinoma (ESCC) metastasis by stabilizing the protein Snail. In GC cells, OTUB1 stabilizes GPX4 by inhibiting its ubiquitination, ultimately promoting GC metastasis by inhibiting ferroptosis (Zhou et al., 2018). This led us to speculate whether the ubiquitination modification of GPX4 by elemene is related to OTUB1 (Li et al., 2023). In this study, OTUB1 stabilizes GPX4 in radioresistance GC cells through deubiquitination. This interaction is crucial for maintaining cellular redox homeostasis and resisting cell death, making it a significant target for therapeutic intervention.

The significance of this research lies in uncovering a novel mechanism through which β-elemene enhances radiosensitivity in GC cells by inducing ferroptosis. This newly identified pathway differs from previously known effects of β-elemene, such as inducing apoptosis and causing cell cycle arrest (Liu et al., 2015). Consequently, the study expands the scientific community’s comprehension of the diverse roles of β-elemene in cancer treatment, particularly regarding radiotherapy, and underscores its potential for inclusion in therapeutic regimens. The findings indicate that β-elemene disrupts the GPX4-OTUB1 axis, likely through allosteric modulation or competitive inhibition (Zhao et al., 2023). This interference requires further structural and biochemical analysis to elucidate the precise mechanisms employed by β-elemene. Gaining such detailed molecular insights will aid in developing targeted therapies that exploit the reliance of radioresistant GC cells on the GPX4-OTUB1 interaction.

## Data Availability

The raw data supporting the conclusions of this article will be made available by the authors, without undue reservation.
